# Editorial: Value-added products from agro-industrial residues by biological approaches

**DOI:** 10.3389/fbioe.2022.990004

**Published:** 2022-10-21

**Authors:** Zhi-Peng Wang, Shang-Yong Li, Xiao-Yan Liu, Jun Xia

**Affiliations:** ^1^ School of Marine Science and Engineering, Qingdao Agricultural University, Qingdao, China; ^2^ School of Basic Medicine, Qingdao University, Qingdao, China; ^3^ Jiangsu Collaborative Innovation Center of Regional Modern Agriculture & Environmental Protection, Huaiyin Normal University, Huaian, China

**Keywords:** value-added products, agro-industrial residues, biological approaches, fermentation, enzyme

Agro-industrial residues at low costs are generated in a considerable amount along the whole chain from harvesting to deep processing. Developing high value-added products with these residues instead of costly food materials has been recommended recently and is supposed to be of increasing economic importance. However, few processes based on biological means are proved feasible economically at present. Optimizing the methods of pretreating different residues or integrating their pretreatment and the subsequent bioprocess is capable of making the value-added production more efficient. From another perspective, creating strategies for the recovery of bioactive molecules from agro-industrial residues is critical to the sustainable development of a bio-based economy.

**FIGURE 1:**
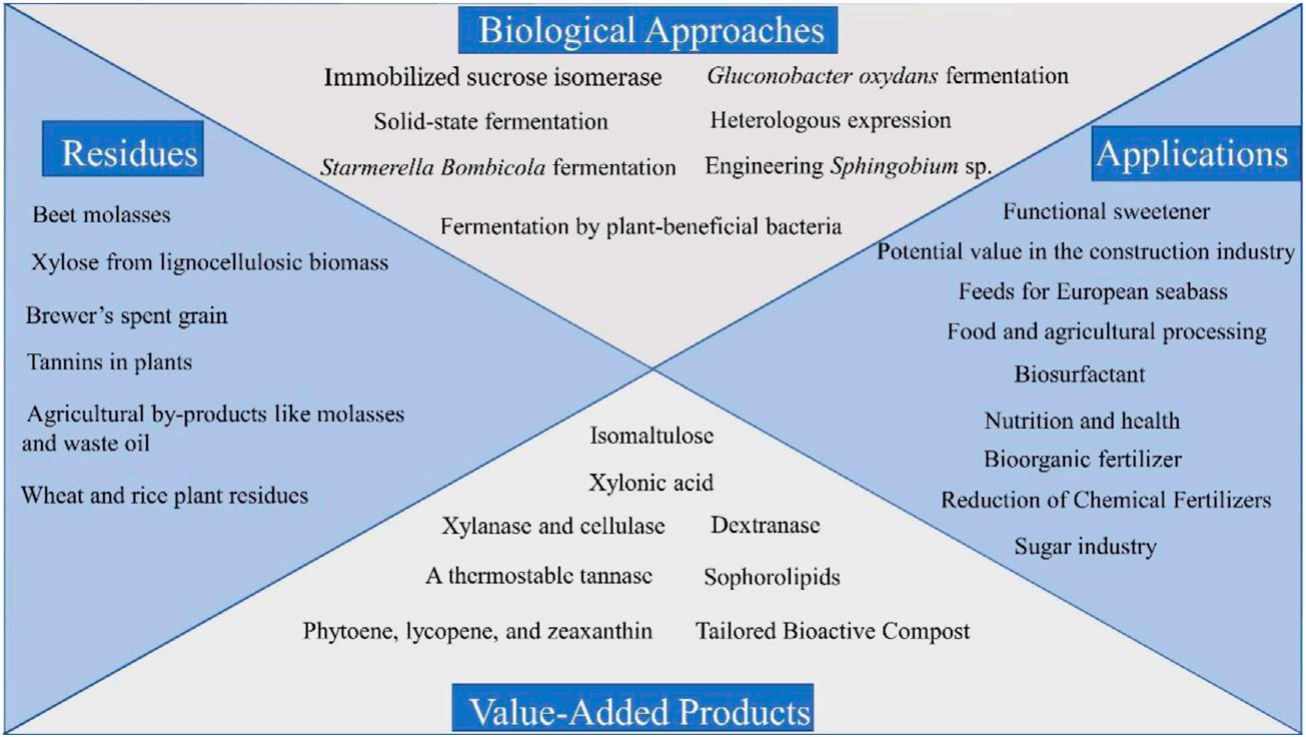
A general categorization of the keywords of the ten research articles.

This Research Topic encompasses ten original research articles and two review articles focusing on this field. Each article shares a remarkable insight into agro-industrial residues, construction of bioprocess, and/or potential applications. The Research Topic centers on the advance of enabling biological approaches and the discovery of functional components (Figure 1). It is expected to see that some descriptions in the Research Topic can present numerous integrated strategies and demonstrate their practical applications to yield value-added products.

Generally, fermentation is the first choice for residue processing. Value-added bioactive compounds can be produced by fermentation using various microorganisms. The fermentation product becomes a participant in microbial cell metabolism or is extracted under the microorganism’s action. Scholars have designed different fermentation routes to maximize the conversion efficiency of the residues. *Aspergillus ibericus* has been used in the solid-state fermentation (SSF) of brewers’ spent grain, which is a fungus without mycotoxin production and has good performance in hydrolyzing the lignocellulosic matrix of agro-industrial residues. Being rich in xylanase and cellulase, the crude extract can serve as an enzyme supplement to aquafeed given to European seabass (*Dicentrarchus labrax*) juveniles, thereby playing a promoting role in feed and protein utilization (Fernandes et al.). Imran et al. proposed a feasible strategy to produce composted organic fertilizer with wheat and rice crop residues and enriched the compost with plant-beneficial bacteria. They found tomato and chili pepper had better growth and a higher yield in the presence of bioactive compost. Thus, an effective way to manage rapidly increasing farm waste is to convert it into compost.

Submerged fermentation based on engineered strains derived from residues has been reported feasible for the bioproduction of chemicals. Liu et al. engineered *Sphingobium* sp. to enrich various carotenoids from common agro-industrial residues, such as soybean meal, soy pulp, and corn steep liquor. Astaxanthin, a member of the carotenoid family, has been biosynthesized at high titer with *Sphingobium* sp., which highlights the potential of microorganisms as resources of industrial importance. Another study indicates that transforming the *Vitreoscilla hemoglobin* gene into *S. bombicola* is conducive to alleviating oxygen limitation (Li et al.). The findings provide evidence to enhance the efficiency of oxygen utilization by *S. bombicola* in the production of SLs on an industrial scale with agro-industrial residues (e.g., molasses and waste oil) as fermentation feedstock.

Notably, agro-industrial residues with unique composition in two articles contribute to efficient bioproduction (He et al.; Wang et al.). With high sucrose content, beet molasses acted as the substrate for isomaltulose production. Immobilized SIase showed good reusability in repeated batch reactions of converting beet molasses pretreated under optimal conditions into isomaltulose. The sucrose conversion reached 97.5% in the first batch and was still higher than 94% after 11 batches (Wang et al.). The process is effective and promising for the industrial production of the functional sweetener isomaltulose. Xylose in a large amount fails to be metabolized and fermented efficiently because lignocellulosic biorefinery is subjected to strain limitations. Therefore, xylonic acid with great value in the construction industry is selected as a substitute for xylose in biorefinery. However, low productivity poses a challenge to xylonic acid fermentation. For this reason, response surface methodology was employed to optimize the specific productivity of xylonic acid. The study made clear the effects of three reaction parameters (biomass concentration, agitation, and aeration) on the production of xylonic acid by *Gluconobacter oxydans* and optimized key process parameters (Wang et al.). These results can provide reference for the efficiency improvement of biotransforming xylose into xylonic acid.

Lemes et al. overviewed the main bioactive components of agro-industrial residues and their biological extraction strategies. The researches provide information to broaden the applications of these bioactive components, especially in the pharmaceutical and food industries. Ma et al. reviewed the production of gluconic acid (GA) and its derivatives through microbial fermentation. They introduced the substitution of agro-industrial residues for carbon sources and integrated routes involving cascade hydrolysis, genetically modified strains, and/or micro- and nanofiltration with a membrane. The review outlines recent state-of-the-art progress, points out existing challenges, and puts forward the development direction of GA production.

Some reports reveal that tannins are partially responsible for the low food intake, growth rate, fodder utilization rate, and protein breakdown of laboratory animals. A study characterized a thermostable tannase sourced from *Aureobasidium melanogenum* T9 and probed into its secretory expression, which was found to be a potential candidate for agricultural and food processing (Liu et al.). Dextran has received increasing attention as a primary pollutant found in sucrose production and storage. Since the hydrolysates of dextranase feature excellent thermal stability and simplicity, it has become a promising enzyme to remove dextran and yield isomaltotriose in the sugar industry (Liu et al.). Besides, both the microbial responses and fungal inhibition of agricultural soil pathogen to the reduction of chemical fertilizers were studied (Shen et al.; Shen et al.).

In conclusion, the valorization of agro-industrial residues helps build a bright future for a bio-based economy. The articles in this Research Topic are believed able to provide much contemporary and valuable information advantageous to the advancement of industrial biotechnology.

